# Structural Damage Identification of Composite Rotors Based on Fully Connected Neural Networks and Convolutional Neural Networks

**DOI:** 10.3390/s21062005

**Published:** 2021-03-12

**Authors:** Veronika Scholz, Peter Winkler, Andreas Hornig, Maik Gude, Angelos Filippatos

**Affiliations:** 1 Center of Information Services and High Performance Computing (ZIH), Technische Universität Dresden, 01187 Dresden, Germany; veronika.scholz@tu-dresden.de (V.S.); peter.winkler1@tu-dresden.de (P.W.); 2 Institute of Lightweight Engineering and Polymer Technology (ILK), Technische Universität Dresden, 01307 Dresden, Germany; andreas.hornig@tu-dresden.de (A.H.); maik.gude@tu-dresden.de (M.G.); 3 Dresden Center for Intelligent Materials (DCIM), Technische Universität Dresden, 01069 Dresden, Germany

**Keywords:** dense neural networks, convolutional neural networks, composites, fully connected neural networks, composite rotors, structural health monitoring (SHM), machine learning

## Abstract

Damage identification of composite structures is a major ongoing challenge for a *secure* operational life-cycle due to the complex, gradual damage behaviour of composite materials. Especially for composite rotors in aero-engines and wind-turbines, a cost-intensive maintenance service has to be performed in order to avoid critical failure. A major advantage of composite structures is that they are able to safely operate after damage initiation and under ongoing damage propagation. Therefore, a robust, efficient diagnostic damage identification method would allow monitoring the damage process with intervention occurring only when necessary. This study investigates the structural vibration response of composite rotors by applying machine learning methods and the ability to identify, localise and quantify the present damage. To this end, multiple fully connected neural networks and convolutional neural networks were trained on vibration response spectra from damaged composite rotors with barely visible damage, mostly matrix cracks and local delaminations using dimensionality reduction and data augmentation. A databank containing 720 simulated test cases with different damage states is used as a basis for the generation of multiple data sets. The trained models are tested using k-fold cross validation and they are evaluated based on the sensitivity, specificity and accuracy. Convolutional neural networks perform slightly better providing a performance accuracy of up to 99.3% for the damage localisation and quantification.

## 1. Introduction

Composite materials become a state-of-the-art material of choice in the structural design of many critical applications where mass and acceleration play an important role. An important advantage beyond the specific stiffness and strength of composite materials is that they are characterized by a gradual damage behaviour and can be safely operated after damage initiation. It is, therefore, all the more important that health monitoring systems prevent the case of catastrophic failure and are able to detect, localise and quantify the damage.

Our goal is to be able to increase the efficiency of composite structures by providing simple and robust structural health monitoring methods by using structural dynamics and machine learning algorithms such as artificial neural networks (ANNs), and specifically fully connected neural networks and convolutional neural networks.

### 1.1. State-of-the-Art

Structural health monitoring (SHM) is an online damage identification process which has been implemented for a wide range of practical applications such as high buildings, bridges, dams, tunnels, and wind turbines [1,2,3,4,5,6]. By monitoring and collecting data from sensors about the current state of the system, anomalies are detected and further structural or economic damage can be averted. In this context, machine learning (ML) methods become increasingly important as they process a large amount of data collected by sensors and identify hidden patterns in the structural complexity of the underlying processes.

In SHM and damage identification systems, sensors collect data from the corresponding component, which are subsequently analyzed to identify the existence of damage, its location and its size. Recently, the combination of powerful computers and their applications to large data sets has lead to several approaches using ML in SHM such as regression [7], k-nearest neighbors [8], decision tree [6], support vector machines [9] and hidden Markov models [10,11].

The use of ANNs in this work is motivated by the complexity of damage detection as there are numerous types and locations of the damage within the same structure. In [12] it has been shown that fully connected neural networks (FC) can classify whether carbon-fibre composites are damaged or undamaged. Therefore, ultrasonic Lamb wave data were preprocessed by outlier and wavelet analysis. Other authors reported the use of feedforward-backpropagation networks as a classification method for damage identification (localisation and size) [13,14,15].

Convolutional neural networks (CNN) are a subclass of ANNs and effectively handle image based data. CNNs are used successfully to identify delamination in carbon fibre-reinforced polymer composites with image data from x-ray tomography [16]. Furthermore, 1-dimensional CNNs are applied to vibration data in time and frequency domain to classify them [17].

Preprocessing of data is an essential step towards a successful training of an ANN. Often, data consists of correlated and therefore redundant features. Dimensionality reduction methods are therefore a valuable tool. Several well-known methods are commonly used to reduce the number of features: linear discriminant analysis (LDA) is well known among the supervised feature reduction methods [18,19]. Principal component analysis (PCA), which transforms a set of correlated variables to a smaller one of uncorrelated variables, has been frequently used in the past. For example Zang and Imregun [20] applied PCA on frequency response functions (FRF) instead of using the raw FRF data as input for the ANN. Kernel PCA (KPCA), which is a nonlinear dimensionality reduction method, can be helpful if it is necessary to handle nonlinear properties of the data [9]. Furthermore, a CNN is capable of merging the two steps of feature reduction and classification into the processes of training and inference as it will enhance the overall network performance [17]. Another aspect concerns the amount of training data: It is largely accepted that large training data makes a model numerically stable and robust and prevents overfitting. Overfitting can occur e.g., when working with small data sets. The practice of synthetically expanding the data set is called data augmentation; several techniques are proposed in [21].

### 1.2. Aim and Outline of the Paper

In this paper, we report on the performance of artificial neural networks for damage identification at composite structures based on simulated vibration signals typical for technical diagnosis of systems. Composite structures can be operated safely even under ongoing damage propagation. It is important to identify the damage process with a robust and efficient diagnostic tool and intervene only when necessary. The aim of the paper is to identify, localise and quantify barely visible damages, mostly matrix cracks and local deliminations in composite rotors by training FCs and CNNs on vibration response spectra. To achieve this, a databank from 720 simulated test cases with different damage states is used as basis for the generation of multiple data sets [22]. The trained models are tested and evaluated based on their sensitivity, specificity and accuracy.

In Section 2, the data sets are presented and four different classifiers with their labels are introduced, each handling a specific problem of damage identification: the identification of (i) radial position, (ii) angular position, (iii) load magnitude and (iv) extent of damaged structures. The damage originates from an initial damage from out-of-plane load and is increased by a propagating damage from in-plane load similar to [23]. Moreover, the data preprocessing techniques i.e., data augmentation is described in Section 2.2 and dimensionality reduction methods are explained in Section 2.4. The introduction of the models can be found in Section 2.5.

In Section 3, the results are presented for every identification level and the classification accuracy is calculated. For the evaluation of the presented damage identification method, a diagnostic performance comparison is conducted for multiple evaluation criteria for both FCs and CNNs. In Section 3.1 the two different network types are compared. The influence of the reduced dimensionality is explained in Section 3.2 and the results with an augmented data set are described in Section 3.3. All results are discussed in Section 4.

## 2. Materials and Methods

This study is based on a data set from numerically generated damage scenarios by using finite element (FE) analysis [22]. It provides different sequences of damage states of composite rotors. Every sequence consists of an initial impact load and then centrifugal load, inflicting initiation of damage and the resulting propagation, respectively. In total 720 damage configurations were generated. The investigated structure is a rotor disc, which is frequently used for basic research investigations regarding the gradual damage behaviour under complex loading cases. It is manufactured from epoxy-reinforced glass fibres, with a thickness of 4 mm and a radius of 250 mm. The results of each simulation are the calculated damage state of the rotor and the corresponding power spectral density (PSD). Different class labels are derived from the damage configurations. The labeled PSDs are used for network training, testing and evaluation [24].

### 2.1. Investigated Composite Rotors

**Figure 1:**
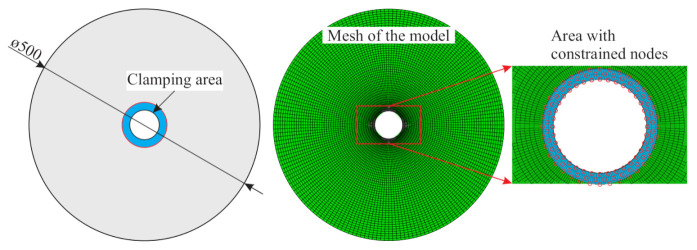
A schematic illustration of the rotor geometry is shown on the left. In the middle, the generated FE-mesh is presented with one element in the thickness direction and an approximate element thickness of 5 mm. On the right, the boundary conditions are illustrated.

In [23], a parametric FE model was developed that enables the generation of multiple loading scenarios to calculate the resulting structural damage as well as the response. For this, the composite failure mode concept of Cuntze [25] was implemented by a user-defined field subroutine (USDFLD) for the implicit solver of the FE-software ABAQUS. As a result, the region for damage initiation is identified and subsequently a reduced stiffness is assigned to mimic damage propagation. In contrast to the typically applied approaches within explicit solving schemes to incorporate non-linear material behaviour, strain rate effects and crack propagation, the proposed modus operandi provides a very efficient way to conduct a large number of simulations with sufficient accuracy [26,27]. The model is meshed using continuum three-dimensional, 8-node linear brick solid elements of type C3D8I, with a total number of 15,500 nodes and 7500 elements. As discussed in [23] the model is representative for the real rotor composite structures. A schematic illustration of the rotor geometry and the FE-mesh is displayed in Figure 1.

The parameterized model setup enables the generation of different positions and magnitudes of the impact load with the subsequent damage states. A full factorial design was applied to derive the relevant FE models, so that the resulting data set contains every possible combination of the chosen parameters. The selected factors were the position, defined as angle and radius, and magnitude of the impact load. Moreover, the linear decrease of the in-plane-load contributes to the simulation. All factors are summarized in Table 1.

Based on the design of experiments (DOE), there are 72 damage propagation scenarios with 10 damage states. In total, this results in 720 damage states for which the power spectral density is calculated in each case. Each scenario takes approximately 5 min to be computed in an Intel(R) Core(TM) i7-2600 CPU @ 3.40 GHz, 3.80 GHz, with an installed RAM of 16.0 GB at a 64-Bit Windows 10 System. A total of 60 h (2.5 days) is required to compute all 720 damage cases. Parallelization techniques on multiple CPUs can be applied to decrease the overall time. Each spectrum covers a frequency range of 0 to 1000 Hz with a frequency resolution of 0.2 Hz resulting in 5001 samples. The simulation of the power spectral density for the undamaged case was not calculated.

### 2.2. Data Set Creation and Data Augmentation

**Figure 2:**
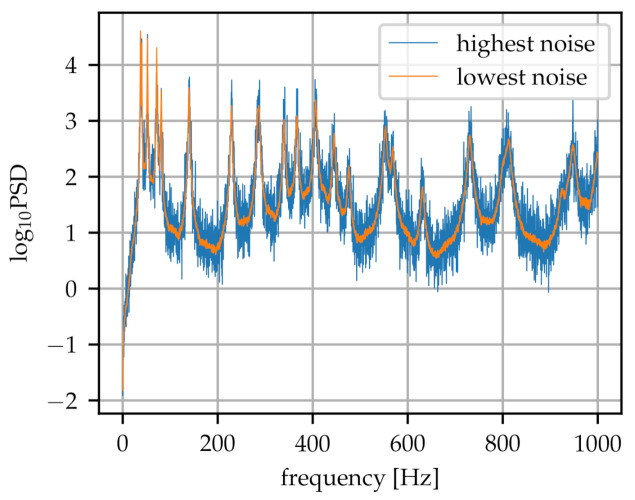
An example spectrum with maximum (factor 0.06) and minimum (factor 0.006) added noise.

A preprocessing step on the data set has been added to the procedure. The PSD ranges from $10^{1}$ as baseline up to $10^{4}$ as peak height, resulting in high changes in the spectra, shown in Figure 2. Therefore, data normalisation is appropriate for numerical reasons: all spectra are logarithmized to base 10, where the first value (zero) was omitted. The normalised spectra are roughly within the range (−10,30) which leads to a numerically well-behaved training of the ANN.

In addition to the original data set, a second data set was created by increasing the original data bank. A data set of sufficient size and variability is essential when training a neural network. It enables the model to learn a more diverse distributed feature representation and it leads to better generalization. Hence, as a starting point we suppose that a simple data augmentation technique is adequate to match the inaccuracies in real-world experimental data. To this end, each spectrum is cloned ten times and overlaid with different noise levels in the direction of the power spectral density. The noise overlay is created by multiplying a normal distribution with standard deviation corresponding to the maximum height in the original spectrum by a factor ranging from 0.006 to 0.06 as shown in Figure 2. The augmented data set has therefore 7920 samples.

### 2.3. Labeling of the Damage States

**Figure 3:**
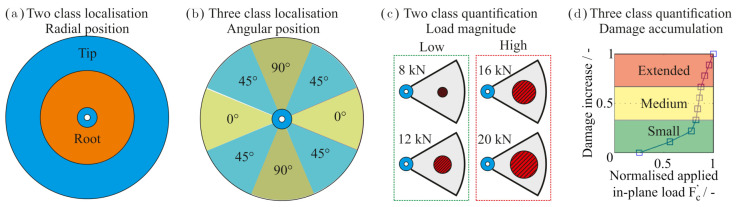
The four physical-based classifiers and their labels. (**a**) shows the radial position, (**b**) the angular position and (**c**) the load magnitude that describes the out-of-plane load. The out-of-plane load with damage accumulation is shown in (**d**) [11].

In total, four classifiers were used for the training. These are the same as described in previous investigations based on the position, type and extent of damage [11]. The discrete values of the factors (position and magnitude of the out-of-plane load plus the in-plane load) were assigned as labels. For that, some of the values are summed up into classes as depicted in Figure 3.

The first classifier referred to as the radial position (RAD) is described by two classes: the rotor tip and root. The second classifier is the angular position (ANG) which results in three classes labelled as “0°”, “45°”, “90°”. The third classifier, load magnitude (MAGN), is quantified by the two classes named “low” and “high”. The fourth classifier describes the speed-dependent damage growth (DAM) in the three classes “Small”, “Medium”, “Extended”. The data were imported into and processed within the Python programming language (version 3.6.9) [28].

### 2.4. Dimensionality Reduction Methods

**Figure 4:**
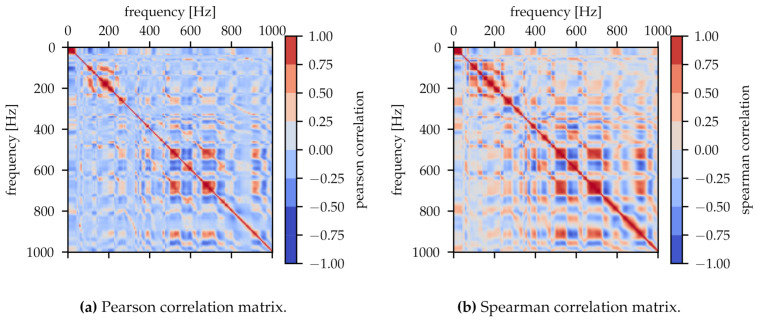
Correlation matrices for 5000 frequencies from the spectrum. (**a**) shows the Pearson correlation matrix for linear dependencies and (**b**) the Spearman correlation matrix for nonlinear dependencies.

To handle the data set of the spectra, each with 5001 spectral density values, a broad neural network is necessary resulting in long training times. In addition, each data point is strongly correlated with its neighbouring points and the network can be successfully trained with fewer data points. Figure 4 shows the Pearson correlation matrix and the Spearman correlation matrix. There are some areas with highly correlated frequencies. Moreover, there is a proportion of nonlinear correlation as can be seen in Figure 4b from the brighter areas compared to Figure 4a.

Because of the underlying correlation in the data, we assumed that it is possible to train the model successfully with fewer spectral density values. Those values are also commonly described as dimensions. Therefore, different methods of dimensionality reduction were tested. A simple approach is reducing the resolution of the frequency spectrum by using only a fraction of all data points, for example every 30th point, and omitting everything else. Other commonly used techniques are principal component analysis and linear discriminant analysis in frequency domain [29]. The goal of PCA is to find components that maximize the variance found in the data. Here it results in 28 or 29 principal components (depending on the train-test split) that explain 90% variance of the total variance of the complete data set. LDA is a supervised method, where the data is projected on one or two axes that best separate the two or three classes. LDA is calculated by solving the generalized eigenvalue problem of the matrix $S_{W}^{-1}*S_{B}$ where $S_{W}$ is the within-class scatter matrix and $S_{B}$ the between-class scatter matrix. The former shows the variation of samples around the same class and the latter the variation between the classes. The difficulty in applying LDA occurs when the data dimension is higher than the number of samples. The rotor data set has 720 samples but 5001 points in frequency domain. This means that the within-class scatter matrix $S_{W}$ becomes singular and the inverse cannot be computed. Therefore, we applied two different methods to reduce dimensionality before LDA is used. The first method is singular value decomposition (SVD). It is implemented to calculate the rank of the data matrix, as reported in [18]. We started with the lowest two frequencies. If the matrix has full rank, the frequencies are kept. We repeated the procedure and dropped variables whenever the rank did not match the number of variables. This leads to a matrix with 96 linear independent frequencies. Another way of reducing dimensionality is applying PCA on the whole data set before using LDA. PCA was implemented with 99.99% variance explanation which leads to 336 principal components. We expected also nonlinear correlation between variables, so we applied KPCA with 29 components. In general, the decline of the model performance was used as the principal criterion to choose the smallest possible number of components (further description in Section 3.2).

### 2.5. Model Training, Validation and Testing

**Figure 5:**
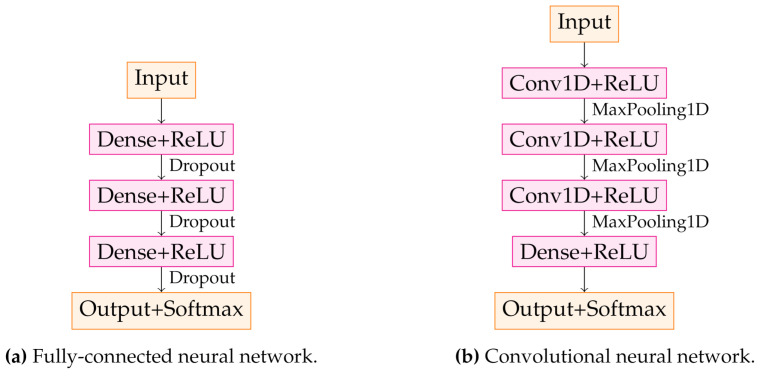
Architecture of NNs used for classification. (**a**) shows the FC. Fully connected (dense) layers were used in combination with the rectified linear activation function (ReLU) and the softmax activation function in the output layer. Dropout layers were inserted to reduce overfitting. (**b**) visualizes the structure of the CNN, with 1D-convolutional and pooling layers being fundamental to this network type.

Two different network types are investigated in this study: a FC and a 1-dimensional CNN. They were trained using Python (version 3.6.9) with Keras (version 2.3.1) and TensorFlow backend as shown in Figure 5. First, the most simple approach is chosen, which is a single-layer neural network. Next, we optimized the training performance by adding hidden layers and tuning hyperparameters by a combination of grid search and manual search. The final FC consists of 5 layers, where the input layer corresponds to the number of neurons. The input layer has 5000 in the case of pure data and a lower number in the case of dimensionality reduction. For the methods in combination with LDA, the models are reduced to a simple logistic regression, which means that they have only one dense layer corresponding to the output layer, as shown in Figure 5a.

The 1-dimensional CNN has been used because the points of the spectra have a distinct order which can be regarded as ’quasi’-time axis (Hz = 1/s). This is not taken into account by a FC, which treats each point of the spectrum individually. A CNN, however, can take into account the order of the data points with ascending frequency. On the other hand, a CNN fulfills two major tasks simultaneously during a single training block: feature extraction and classification. This ability provides an improved classification performance and efficiency. The CNN has three blocks consisting of a convolutional layer followed by a pooling layer, as shown in Figure 5b. Then the flattened output is transmitted to a dense layer followed by the output layer with binary neurons leading to multi-class classification.

Before the training of the models, the data was split into three parts: training, validation, and test sets. We applied a stratified 5-fold cross validation for the test phase. During five rounds each subset is used once as the test set and the remaining subsets as training data. Here, the original distribution of classes is transferred to the test set to reflect the deterministic process of the data generation process. We did not take into account the structure of all different combinations as depicted in Table 2 assuming that the spectra are sufficiently similar. The training data is again split into a validation set (20%) and the actual training set (80%). The validation set is crucial to give an estimate of the model’s performance. These validation results are quality measures for the choice of the hyperparameters and the final selection of the model.

Hyperparameters in Table 2 were tuned for each classifier separately. After this process, the test data set provides an independent evaluation of the model. The manual optimization was performed by calculating the confusion matrix with accuracy and sensitivity as target functions. The latter was chosen from an engineering point of view in order to most effectively detect all possible forms of damage of the rotor to prevent further propagation and catastrophic failure while accepting the possibility of false alarms (false positives).

### 2.6. Overview of the Generated Machine Learning Models

The different models were trained from a configuration of the following parameters:

1. Algorithm: FC, 1D-CNN,
2. Data set: original (OD) or augmented (AD),
3. Dimensionality reduction method: pure data, each 30th frequency, PCA, PCA+LDA, SVD+LDA and KPCA,
4. Classifier: radial position (RAD), angular position (ANG), load magnitude (MAGN) and damage accumulation (DAM).

The parameters used for the model training are displayed in Table 2, resulting in 36 different models. We discarded the combination of CNN and dimensionality reduction methods because the CNN itself includes a form of feature extraction as described in Section 2.5. All models and their names and results can be looked up in Table A1. We display the mean accuracy, sensitivity, and specificity as well as the standard deviation for the cross validation results of the final test data set in the table. In the following sections we refer to the different models by their names.

## 3. Results

### 3.1. Classification with Fully Connected Neural Network and Convolutional Neural Network

**Figure 6:**
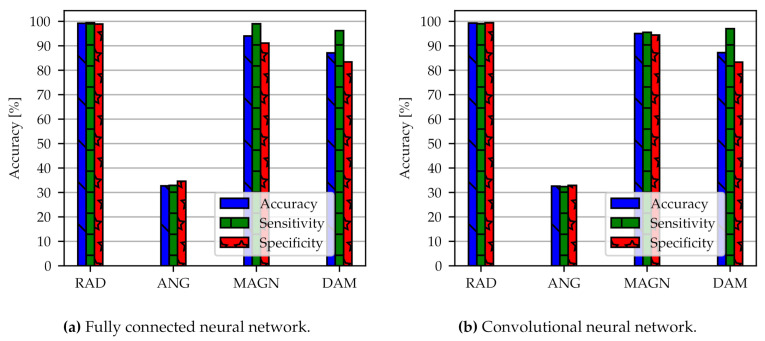
Classification results of two different network architectures FC (**a**) and CNN (**b**). We compare the models FC-OD-Pure-RAD, FC-OD-Pure-ANG, FC-OD-Pure-MAGN and FC-OD-Pure-DAM (**a**) with CNN-OD-Pure-RAD, CNN-OD-Pure-ANG, CNN-OD-Pure-MAGN and CNN-OD-Pure-DAM (**b**). The abbreviations can be looked up in Appendix A. OD stands for original data.

The test accuracy, sensitivity, and specificity of the FC for the four classifiers are shown in Figure 6a (models FC-OD-Pure-RAD, FC-OD-Pure-ANG, FC-OD-Pure-MAGN and FC-OD-Pure-DAM). The simple algorithm of the FC already has high predictive performance and results are roughly at the same quality for each classifier. An exception is the angular position with an accuracy of around 30% which performs no better than a random assignment to the three classes. Radial position has the overall best performance with 99.2% accuracy and 99.4% sensitivity, followed by the load magnitude with 94.0% (99.0% sensitivity) and damage accumulation with 87.1% (96.2% sensitivity). The specificity has lower values than accuracy and sensitivity but is still higher than 80%. Typically we observe this trade-off between sensitivity and specificity when training neural network models. We have tuned the model parameters in such a way that higher sensitivity is preferred over higher specificity. Radial position and angular position in Figure 5 are the limiting cases where the assignment to the classes is exact or random. As a result, there is no more differences between sensitivity and specificity.

From Figure 6b with models CNN-OD-Pure-RAD, CNN-OD-Pure-ANG, CNN-OD-Pure-MAGN, and CNN-OD-Pure-DAM it can be seen that the 1D-CNN shows slightly better predictive performance than the FC. Still, accuracy is comparable with 95.0% (95.5% sensitivity) for load magnitude and 87.2% (97.0% sensitivity) for damage accumulation. In comparison to the FC, the accuracy has improved, particularly for damage accumulation. Again, the best performance can be observed for the radial position with 99.3% accuracy (99.1% sensitivity). The specificity for CNN model is also higher than 80%. The training for angular position was not successful.

Machine learning techniques were explored in the field of composite rotors but ANNs have not been applied to such data structure [11]. In this section, we confirmed that it is possible to use ANNs to predict the radial position, the damage accumulation and the load magnitude from spectral response data. There are only very few cases that were not classified correctly. The FC and the CNN are both appropriate for this task [13,14,15,17].

### 3.2. Influence of Reduced Dimensionality

To compare dimensionality reduction methods it is necessary to choose the number of components, i.e., the number of reduced dimensions.

**Figure 7:**
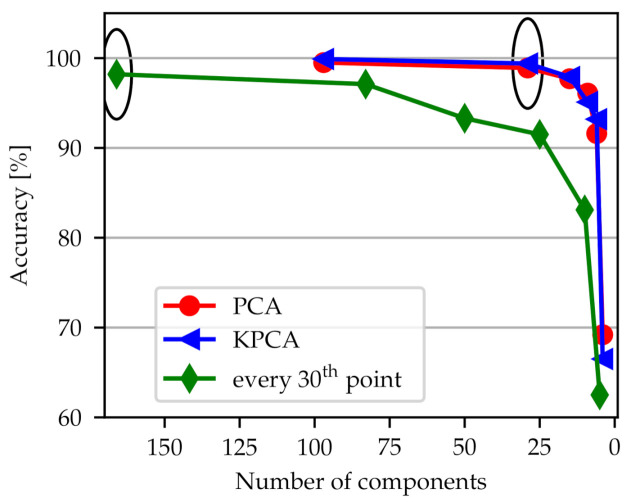
Accuracy for each of the dimensionality reduction methods PCA, KPCA and using only every 30th frequency point depends on the number of components. Representative curves are shown for the classifier detecting the radial position. The encircled points indicate the chosen number of components.

We selected the lowest possible number of components before accuracy starts to drop rapidly as depicted in Figure 7 for the classifier radial position. The simplest dimensionality reduction method is to omit data points (green line). Here, it was chosen to use every 30th frequency point. However, Figure 7 shows that an algorithm for dimensionality reduction performs better than manually decreasing the spectra. In PCA an important input parameter is the percentage of the variance of the original data that is explained by the reduced data. We have chosen this explained variance to be 90% resulting in 29 components. In KPCA the same number of components has been chosen because the corresponding curve is nearly identical to the one for PCA. All three choices lead to an accuracy which is above 98% as can be seen from the encircled points in Figure 7.

**Figure 8:**
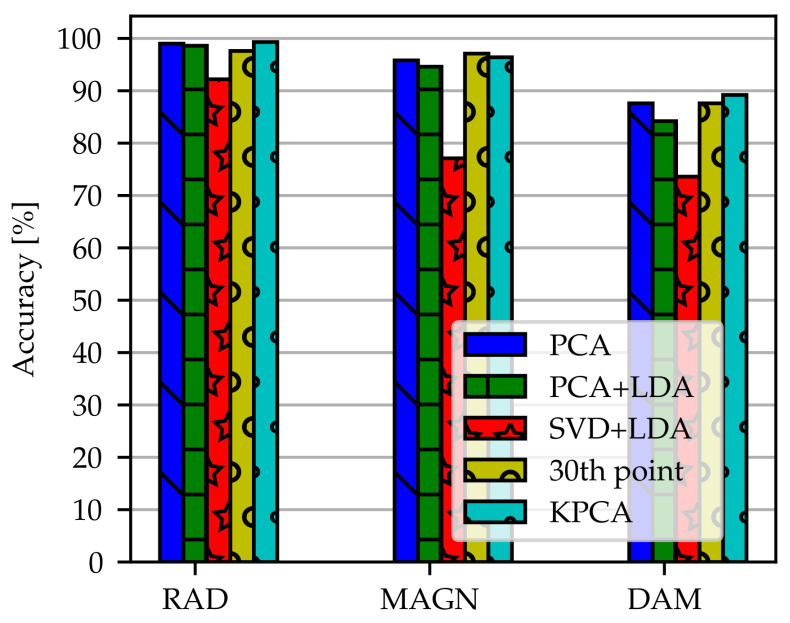
Comparison of dimensionality reduction methods for the models FC-OD-30thFreq-RAD/-MAGN/-DAM, FC-OD-PCA-RAD/-MAGN/-DAM, FC-OD-PCA+LDA-RAD/-MAGN/-DAM, FC-OD-SVD+LDA-RAD/-MAGN/-DAM and FC-OD-KPCA-RAD/-MAGN/-DAM. Accuracy calculated with FC is shown. The dimensionality reduction methods are PCA, PCA+LDA, SVD+LDA, KPCA and using only every 30th data point. Angular position was omitted because there was no training effect at all.

The accuracies obtained by dimensionality reduction methods with the models FC-OD-30thFreq-RAD/-MAGN/-DAM, FC-OD-PCA-RAD/-MAGN/-DAM, FC-OD-PCA+LDA-RAD/-MAGN/-DAM, FC-OD-SVD+LDA-RAD/-MAGN/-DAM and FC-OD-KPCA-RAD/-MAGN/-DAM are summarized in Figure 8. In some cases the number of components used as input could be reduced dramatically without loosing much performance. It can be seen that PCA, Kernel PCA, using every 30th data point and LDA in combination with PCA have nearly the same height. They clearly outperform LDA with SVD. In relation to the number of components PCA and KPCA are the most effective reduction methods since they use only 29 components as input to the network. In our case, KPCA has no advantage over PCA. It should be noted that accuracy does not decrease compared to the training with original spectra except for data reduced by SVD+LDA. Specificity and sensitivity can be extracted from Table A1. There is no major discrepancy between these performance measures and overall accuracy.

Dimensionality reduction methods are studied extensively [30]. Nevertheless, it depends on the data itself if a technique is applicable and leads to good results. There are several advantages of reducing the input data dimension as for example a decreased training time [20]. In this section, the application of dimensionality reduction methods is investigated and we show that it is not necessary to use the full spectrum.

### 3.3. Influence of Synthetically Augmented Data Set

**Figure 9:**
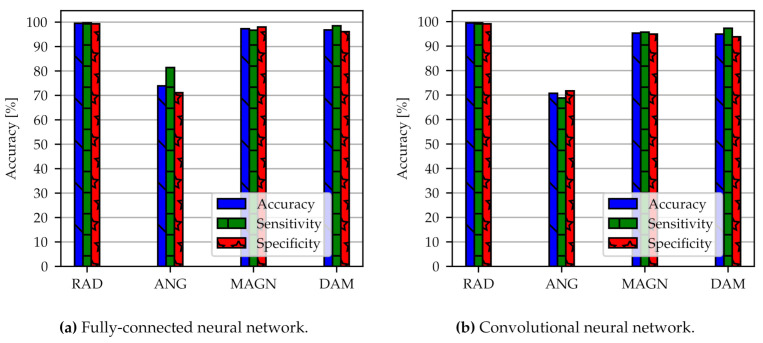
Classification results with an augmented data set for the two network architectures: FC (**a**) with models FC-AD-Pure-RAD/-ANG/-MAGN/-DAM and CNN (**b**) with models CNN-AD-Pure-RAD/-ANG/-MAGN/-DAM. The data set contains in total 7920 spectra. Three bars corresponding to accuracy, sensitivity and specificity are shown.

An augmented data set with a higher number of samples but without dimensionality reduction leads to an increase of accuracy compared to the original data set as reported in Figure 9. Here, the models FC-AD-Pure-RAD/-ANG/-MAGN/-DAM and CNN-AD-Pure-RAD/-ANG/-MAGN/-DAM are applied to the data. Radial position, load magnitude and damage accumulation achieve an accuracy as well as a sensitivity and specificity above 96%. Angular position accuracy improves from roughly 30% to 70%. The increased accuracy indicates that a large data set makes the model numerically more stable and robust.

Typically, neural networks are trained with thousands of samples [31] but we have only around seven hundred samples available. In the case of experimental data, there are often several recordings of the same composite that are influenced by measurement noise. We intended to simulate this situation also in our synthetic data set with data augmentation techniques. Those data augmentation techniques are commonly used in image classification tasks [32] but are not investigated for vibrational data from composite rotors. We showed that the increase of sample size improves the results of the neural network classification in comparison to the original data set because there are more training examples available for each simulation scenario.

## 4. Discussion and Conclusions

In this paper, we showed that it is possible to detect, localize, and quantify barely visible damages of composite structures with ANNs. It addresses a topic that has not been fully exploited, i.e., the consideration of frequency response spectra as input for ANNs for the prediction of damages. The ANNs are able to classify size and location of an initial damage in composite rotors from vibration response spectra by neural networks correctly. Two different network types were analyzed. We report a test accuracy ranging from 87% to 99% for a simple FC network and a 1-dimensional CNN, where the best performance is observed for the radial position of the damage. While in a FC, each point of the spectra is treated as an individual feature, a CNN takes into account the ordered sequence of the points in the vibration response spectra and the correlations within. This may explain why the CNN yields slightly better results, especially for damage accumulation and load magnitude. Furthermore, the structure of a CNN includes already a feature extraction mechanism so that there is no additional dimensionality reduction technique necessary before the training. As criteria for model and hyperparameter selection, we used the validation accuracy. The test results lie within the range of the standard deviation of cross validation results (see Table A1).

We observe that the ANN approach yields results comparable to the hidden Markov model (HMM) used in [22]. An exception is the angular position, where it was not possible to train the network successfully no matter which model or model parameters were used. We could not clearly determine if this is a problem of the data itself or if the model is not appropriate to classify the angular position. Further investigations with more data, other machine learning models as well as considerations of the underlying physics and descriptive data analysis might lead to a more detailed insight. Furthermore, dependencies between the classifiers can be investigated to gain insights into the role of the angular position.

We implemented several dimensionality reduction algorithms in order to use networks with lower input dimensions. The test accuracy did not decrease when using only every 30th frequency value, which indicates that the spectra contain redundant information for the purpose of damage classification. The most successful dimensionality reduction algorithms were PCA and KPCA with approximately thirty components. The results obtained by using LDA in combination with rank determination via singular value decomposition had lower accuracy compared to using raw data. The combination of LDA with other dimensionality reduction techniques is mandatory since the number of samples is smaller than the number of frequencies. In conclusion we achieve good results with and without dimensionality reduction. Depending on the amount of data of future research and the computational resources both options are reasonable.

As the data set contains only several hundreds of samples and is therefore small for the training of a neural network, we applied a simple data augmentation technique by adding random noise to the samples. This data augmentation increased accuracy and sensitivity to nearly 100% and the corresponding values for angular position increased to 70%. It confirms that the selected models are appropriate methods for classifying damage states of composite rotors based on simulated vibrational data. Moreover, it means that the augmentation technique and therefore the higher sample size is a key factor in evaluating synthetic data of composite rotors. Nevertheless, instead of applying only a random noise term to the original data, it can be more precisely analysed which types of noise occur during the experimental measurement process in order to simulate it more accurately in the artificial data base.

Future research might address the topic of generalizing the model to experimental data. For example, mixture of synthetic and experimental data can be used for the training of the ANN models. It is important to think about the balance of synthetic and experimental data, maybe by providing weights or biases. Further development of physical-based modelling has to be undertaken. In the state-of-the art simulations, typical 2–3% deviations between models and reality are to be expected by composite structures [23]. It is a challenge that the ANN still performs adequately and can still generalise even with this range of deviations in the mixed experimental-numerical data set.

In ML applications, it is common practice to use tens of thousands of samples in a training run. However, one has to keep in mind that the creation of data in engineering science from experimental setups is often time consuming and expensive. This increases the need to handle also small data sets in a way that makes reliable predictions possible. Despite the use of a relatively small amount of data, we have shown that a successful classification of simulated damaged rotors using ANNs is possible with excellent performance. This is a valid starting point for creating experimental data sets and developing ML applications which are also able to perform the damage classification task on real world data.

## Figures and Tables

**Table 1:** All factors that are used for the simulation with unit and levels of the factors.

Factor	Unit	Levels
impact load	kN	8, 12, 16, 20
radius	mm	75, 105, 135, 165, 195, 225
angle	°	0, 45, 90
centrifugal load	%	51, 53, 56, 58, 59, 60, 63, 69, 76, 100

**Table 2:** Hyperparameters used for the networks FC and CNN depending on the four classifiers RAD, ANG, MAGN and DAM.

Parameter	FC-RAD	FC-MAGN	FC-DAM	CNN-RAD	CNN-DAM
	FC-ANG			CNN-MAGN	
				CNN-ANG	
optimizer	Adam	Adam	Adam	Adam	Adam
learning rate	0.0001	0.0001	0.0001	0.001	0.0001
epochs	100	100	100	100	100
batch size	64	64	64	64	16
filter	-	-	-	40, 40, 20	90, 90, 60
kernel size	-	-	-	9	3
pooling size	-	-	-	6	3
dense layer	1000, 100, 100	5010, 1000, 100	6000, 1000, 100	20	20

## Data Availability

The data presented in this study are available on request from the corresponding author.

## Abbreviations

The following abbreviations are used in this manuscript:

ANG	Angular Position
ANN	Artificial Neural Network
CNN	Convolutional Neural Network
DAM	Damage Accumulation
FC	Fully Connected Neural Network
FE	Finite Element
FRF	Frequency Response Function
HMM	Hidden Markov Model
KPCA	Kernel Principal Component Analysis
LDA	Linear Discriminanat Analysis
MAGN	Load Magnitude
ML	Machine Learning
OD	Original Data
PCA	Principal Component Analysis
PSD	Power Spectral Density
RAD	Radial Position
ReLU	Rectified Linear Unit
SHM	Structural Health Monitoring
SVD	Singular Value Decomposition

## Appendix A

**Table A1:** All trained models and combinations of network, data type and dimensionality. Test accuracy, sensitivity, specificity and validation accuracy with cross-validation error is displayed.

Network Type	Data Type	Dimensionality Reduction	Classifier	Name	Acc. [%]	Sens. [%]	Spec. [%]
FC	Original logarithmized data	Pure data	RAD	FC-OD-Pure-RAD	99.2±0.5	99.4±0.6	98.9±0.6
FC	Original logarithmized data	Pure data	ANG	FC-OD-Pure-ANG	32.7±4.9	32.9±7.5	34.6±6.0
FC	Original logarithmized data	Pure data	MAGN	FC-OD-Pure-MAGN	94.0±4.3	99.0±1.5	91.1±7.0
FC	Original logarithmized data	Pure data	DAM	FC-OD-Pure-DAM	87.1±1.4	96.2±2.6	83.4±2.5
FC	Original logarithmized data	Every 30th frequency	RAD	FC-OD-30thFreq-RAD	97.6±1.4	99.2±1.1	96.1±2.5
FC	Original logarithmized data	Every 30th frequency	ANG	FC-OD-30thFreq-ANG	−	−	−
FC	Original logarithmized data	Every 30th frequency	MAGN	FC-OD-30thFreq-MAGN	97.1±1.8	98.4±2.2	96.0±2.1
FC	Original logarithmized data	Every 30th frequency	DAM	FC-OD-30thFreq-DAM	87.6±1.6	92.3±6.3	85.9±2.8
FC	Original logarithmized data	PCA	RAD	FC-OD-PCA-RDA	99.0±0.7	100.0±0	98.1±1.3
FC	Original logarithmized data	PCA	ANG	FC-OD-PCA-ANG	−	−	−
FC	Original logarithmized data	PCA	MAGN	FC-OD-PCA-MAGN	95.8±1.6	95.9±2.7	95.8±2.7
FC	Original logarithmized data	PCA	DAM	FC-OD-PCA-DAM	87.6±2.5	94.2±3.1	84.5±3.9
FC	Original logarithmized data	PCA + LDA	RAD	FC-OD-PCA+LDA-RAD	98.6±0.6	99.4±0.7	97.7±1.3
FC	Original logarithmized data	PCA + LDA	ANG	FC-OD-PCA+LDA-ANG	−	−	−
FC	Original logarithmized data	PCA + LDA	MAGN	FC-OD-PCA+LDA-MAGN	94.6±2.0	97.3±2.0	92.2±2.1
FC	Original logarithmized data	PCA + LDA	DAM	FC-OD-PCA+LDA-DAM	84.2±1.4	93.5±3.8	80.3±2.7
FC	Original logarithmized data	SVD + LDA	RAD	FC-OD-SVD+LDA-RAD	92.2±2.5	91.3±2.7	93.0±3.8
FC	Original logarithmized data	SVD + LDA	ANG	FC-OD-SVD+LDA-ANG	−	−	−
FC	Original logarithmized data	SVD + LDA	MAGN	FC-OD-SVD+LDA-MAGN	77.1±4.5	79.9±3.4	74.9±6.9
FC	Original logarithmized data	SVD + LDA	DAM	FC-OD-SVD+LDA-DAM	73.6±2.7	89.3±5.2	67.6±2.9
FC	Original logarithmized data	KPCA	RAD	FC-OD-KPCA-RAD	99.3±6.2	99.5±1.0	99.2±1.0
FC	Original logarithmized data	KPCA	ANG	FC-OD-KPCA-ANG	−	−	−
FC	Original logarithmized data	KPCA	MAGN	FC-OD-KPCA-MAGN	96.4±1.5	96.8±1.5	95.9±2.5
FC	Original logarithmized data	KPCA	DAM	FC-OD-KPCA-DAM	89.2±3.4	94.3±2.0	86.9±4.5
FC	Augmented logarithmized data	Pure data	RAD	FC-AD-Pure-RAD	99.5±0.4	99.7±0.4	99.3±0.5
FC	Augmented logarithmized data	Pure data	ANG	FC-AD-Pure-ANG	73.9±2.3	81.4±2.5	71.1±3.0
FC	Augmented logarithmized data	Pure data	MAGN	FC-AD-Pure-MAGN	97.3±0.5	96.7±1.2	98.0±0.5
FC	Augmented logarithmized data	Pure data	DAM	FC-AD-Pure-DAM	96.8±0.5	98.5±0.3	96.1±0.7
1D-CNN	Original logarithmized data	Pure data	RAD	CNN-OD-Pure-RAD	99.3±0.6	99.1±0.9	99.4±0.6
1D-CNN	Original logarithmized data	Pure data	ANG	CNN-OD-Pure-ANG	32.6±1.4	32.4±1.4	32.9±1.6
1D-CNN	Original logarithmized data	Pure data	MAGN	CNN-OD-Pure-MAGN	95.0±2.0	95.5±4.2	94.4±3.9
1D-CNN	Original logarithmized data	Pure data	DAM	CNN-OD-Pure-DAM	87.2±3.4	97.0±2.9	83.3±5.3
1D-CNN	Augmented logarithmized data	Pure data	RAD	CNN-AD-Pure-RAD	99.5±0.2	99.5±0.3	99.1±0.2
1D-CNN	Augmented logarithmized data	Pure data	ANG	CNN-AD-Pure-ANG	70.7±4.5	68.8±4.6	71.7±4.6
1D-CNN	Augmented logarithmized data	Pure data	MAGN	CNN-AD-Pure-MAGN	95.3±0.6	95.7±1.2	94.9±1.6
1D-CNN	Augmented logarithmized data	Pure data	DAM	CNN-AD-Pure-DAM	94.9±0.8	97.3±0.3	93.8±1.2
